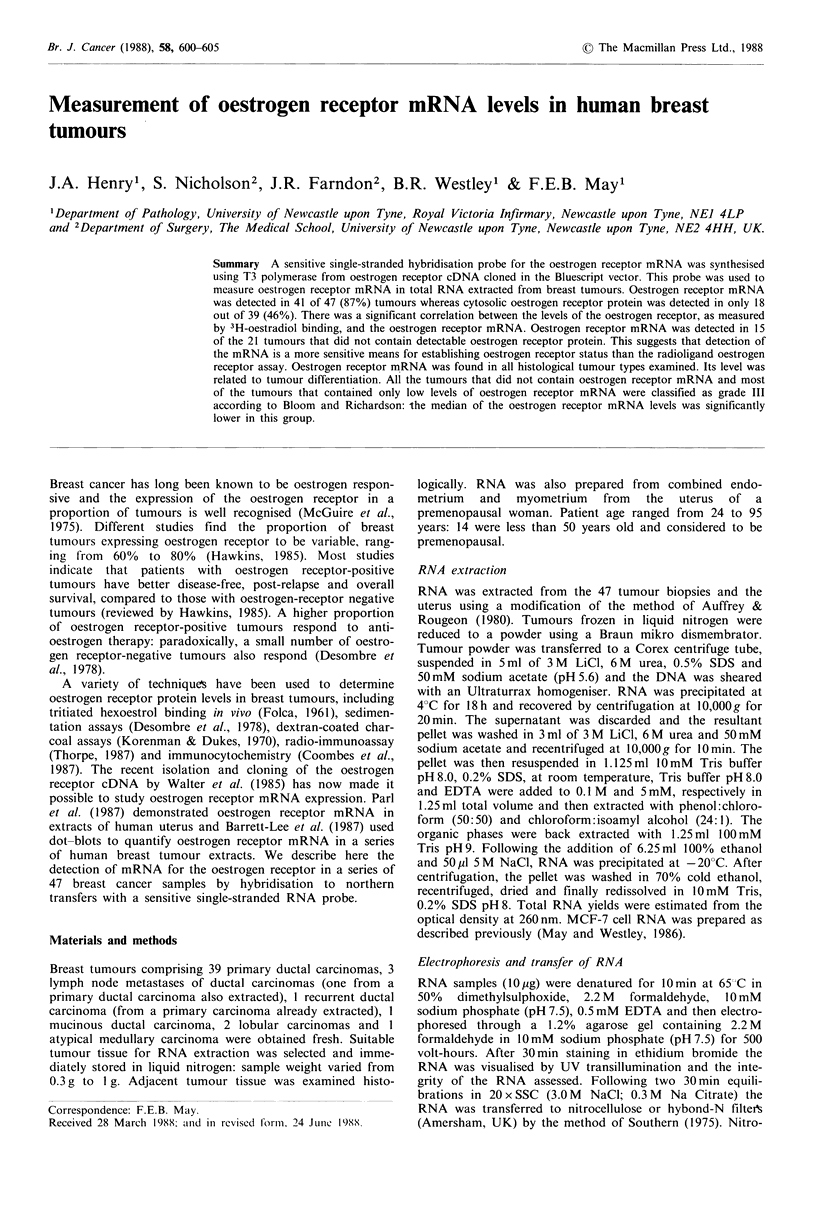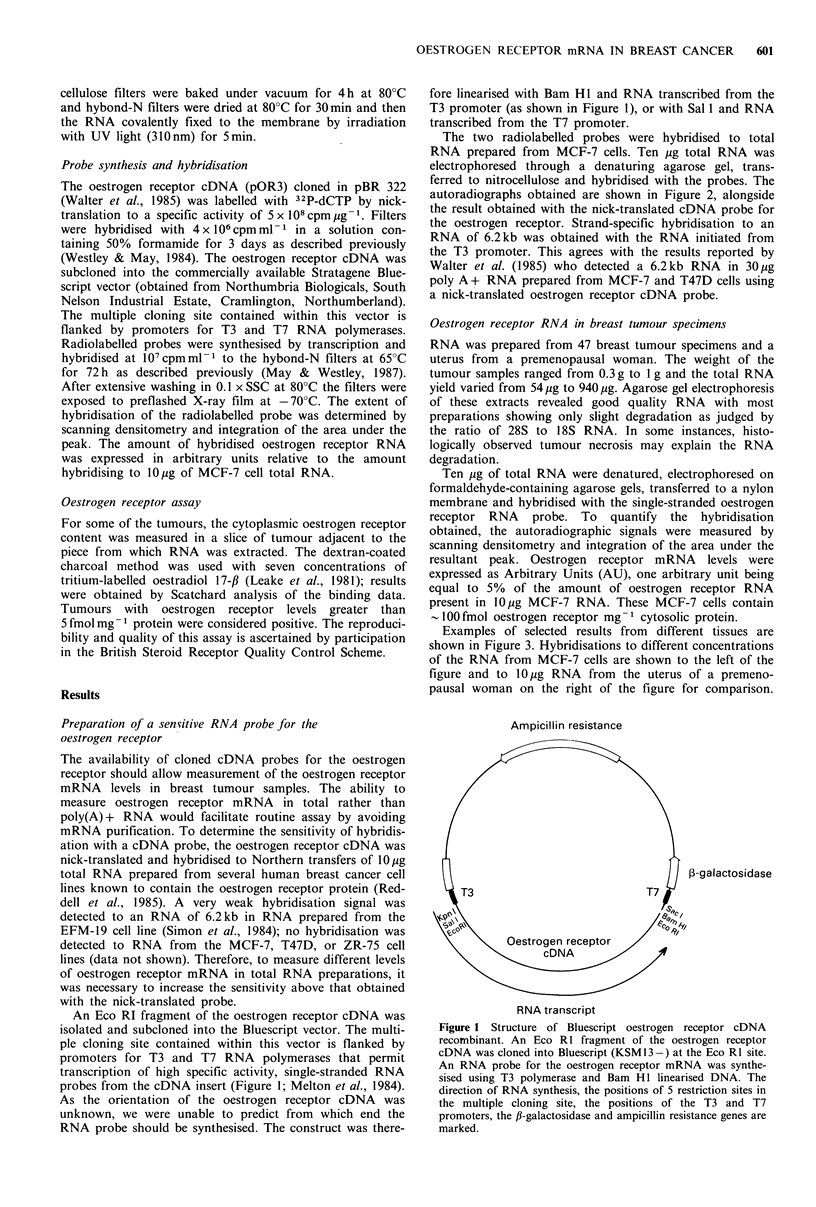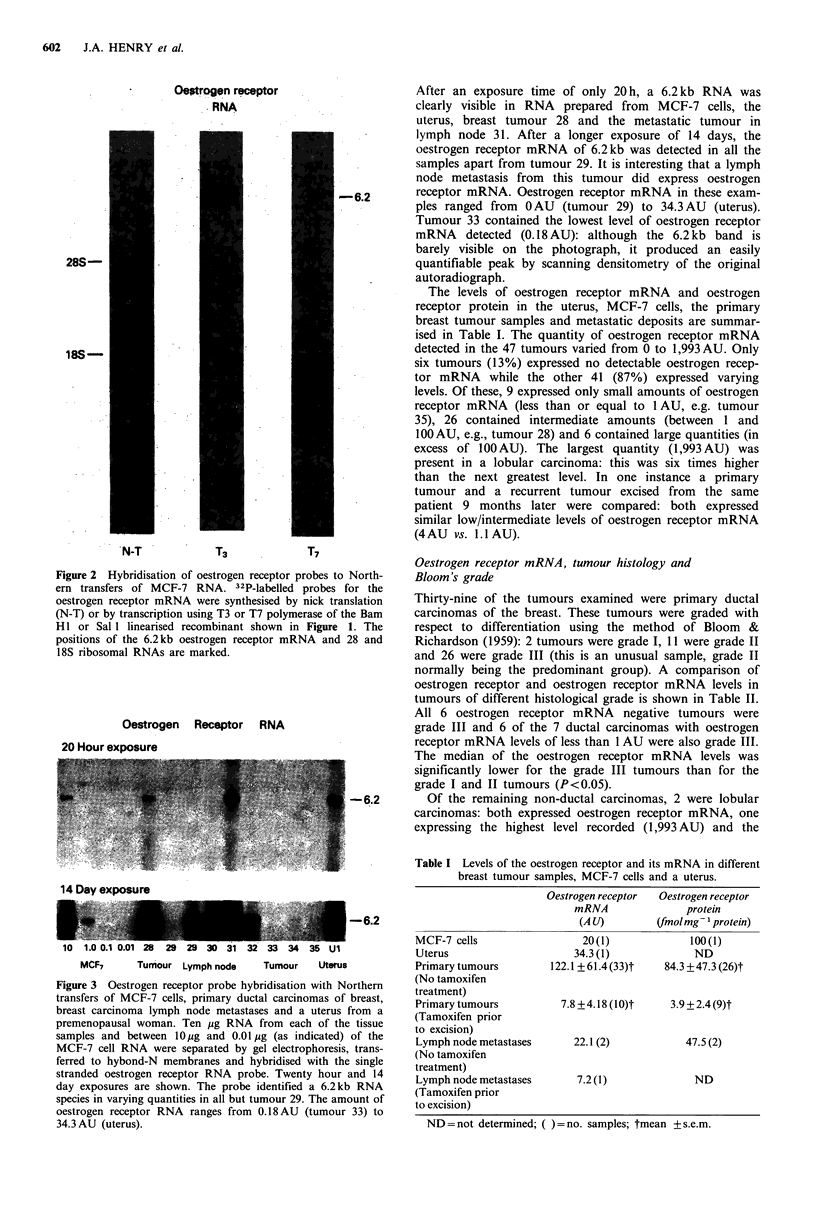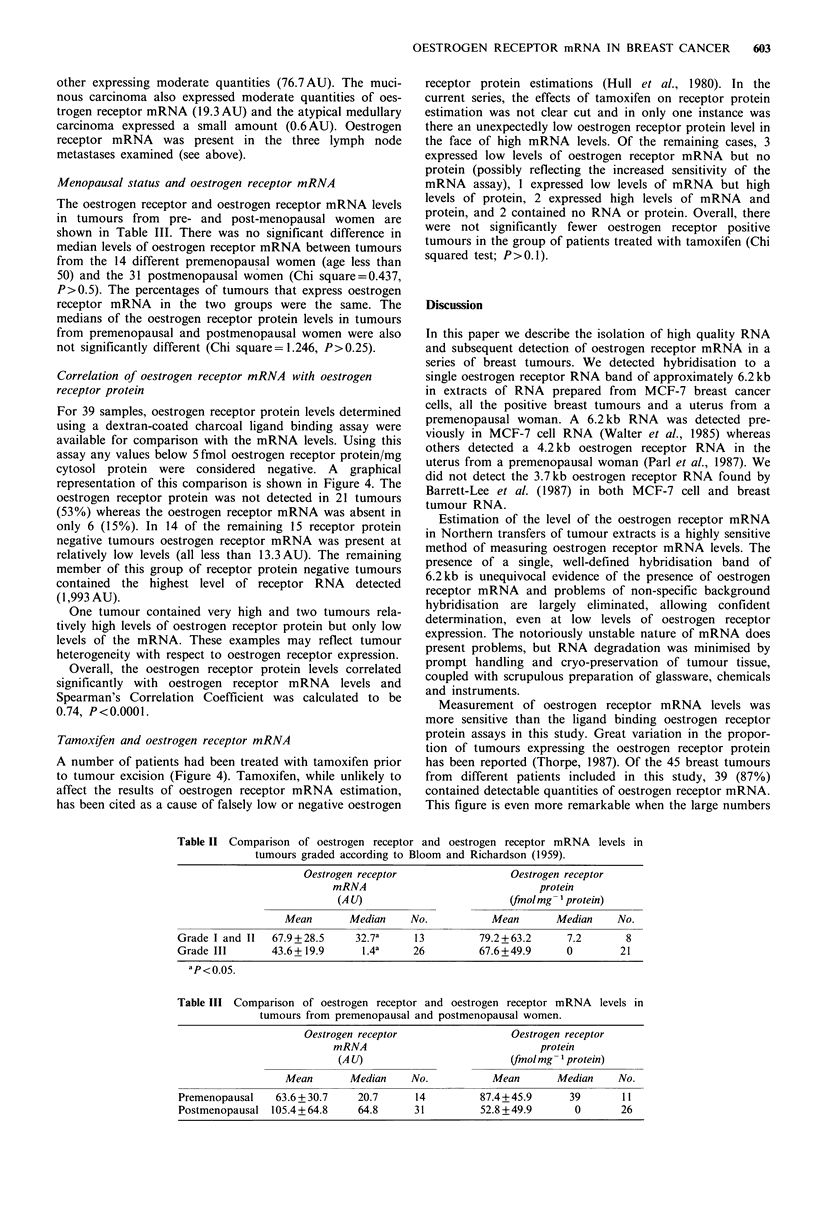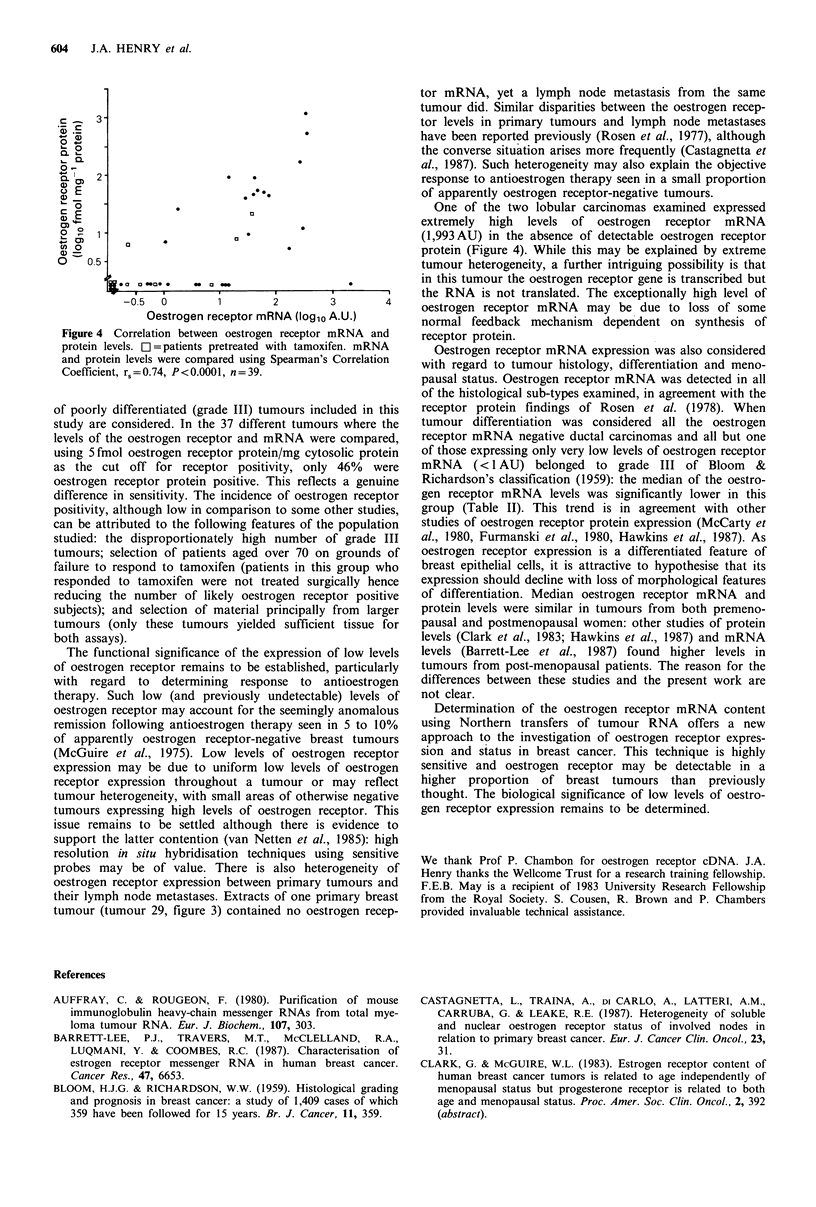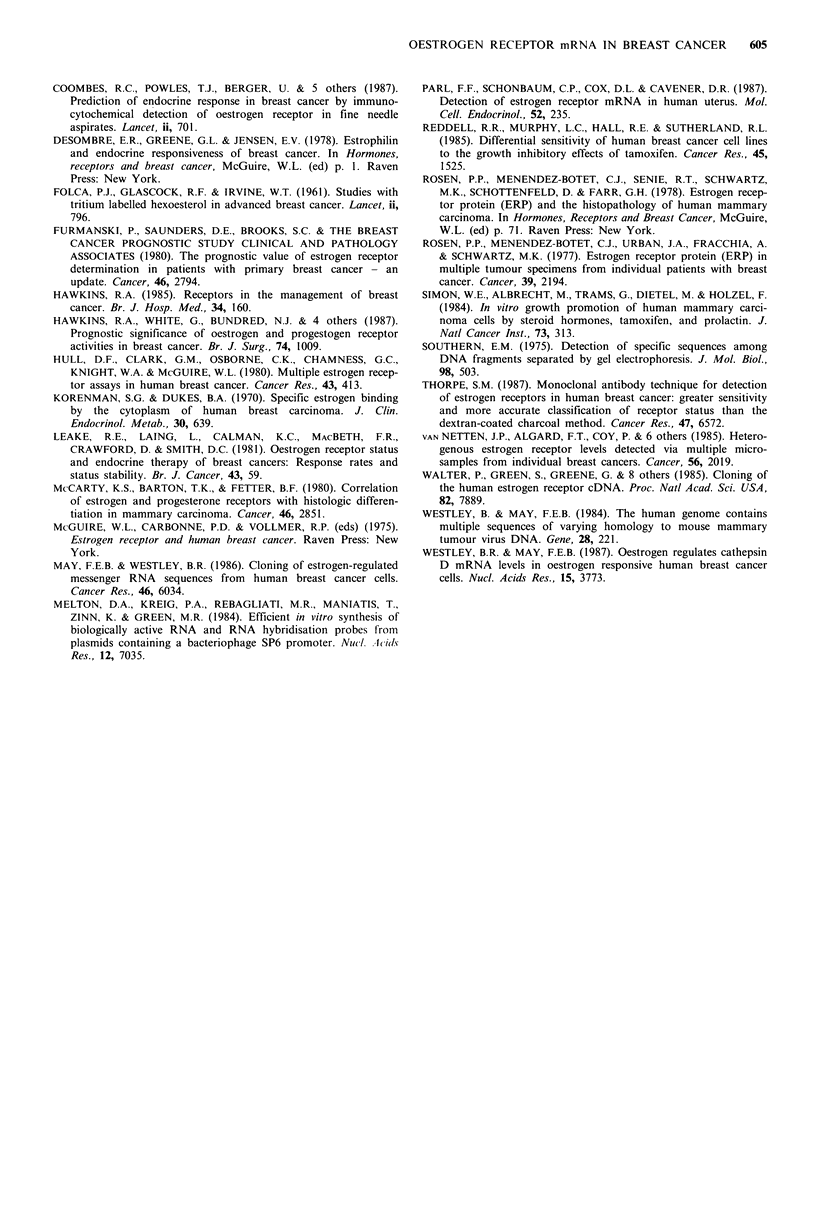# Measurement of oestrogen receptor mRNA levels in human breast tumours.

**DOI:** 10.1038/bjc.1988.267

**Published:** 1988-11

**Authors:** J. A. Henry, S. Nicholson, J. R. Farndon, B. R. Westley, F. E. May

**Affiliations:** Department of Pathology, University of Newcastle upon Tyne, Royal Victoria Infirmary, UK.

## Abstract

**Images:**


*Br. J. Cancer* (1988), **58**, 600–605 © The Macmillan Press Ltd., 1988

### Measurement of oestrogen receptor mRNA levels in human breast tumours

J.A. Henry[1], S. Nicholson[2], J.R. Farndon[2], B.R. Westley[1] & F.E.B. May[1]

[1]*Department of Pathology, University of Newcastle upon Tyne, Royal Victoria Infirmary, Newcastle upon Tyne, NE1 4LP and* [2]*Department of Surgery, The Medical School, University of Newcastle upon Tyne, Newcastle upon Tyne, NE2 4HH, UK.*

**Summary** A sensitive single-stranded hybridisation probe for the oestrogen receptor mRNA was synthesised using T3 polymerase from oestrogen receptor cDNA cloned in the Bluescript vector. This probe was used to measure oestrogen receptor mRNA in total RNA extracted from breast tumours. Oestrogen receptor mRNA was detected in 41 of 47 (87%) tumours whereas cytosolic oestrogen receptor protein was detected in only 18 out of 39 (46%). There was a significant correlation between the levels of the oestrogen receptor, as measured by $^{3}$H-oestradiol binding, and the oestrogen receptor mRNA. Oestrogen receptor mRNA was detected in 15 of the 21 tumours that did not contain detectable oestrogen receptor protein. This suggests that detection of the mRNA is a more sensitive means for establishing oestrogen receptor status than the radioligand oestrogen receptor assay. Oestrogen receptor mRNA was found in all histological tumour types examined. Its level was related to tumour differentiation. All the tumours that did not contain oestrogen receptor mRNA and most of the tumours that contained only low levels of oestrogen receptor mRNA were classified as grade III according to Bloom and Richardson: the median of the oestrogen receptor mRNA levels was significantly lower in this group.

Breast cancer has long been known to be oestrogen responsive and the expression of the oestrogen receptor in a proportion of tumours is well recognised (McGuire *et al.*, 1975). Different studies find the proportion of breast tumours expressing oestrogen receptor to be variable, ranging from 60% to 80% (Hawkins, 1985). Most studies indicate that patients with oestrogen receptor-positive tumours have better disease-free, post-relapse and overall survival, compared to those with oestrogen-receptor negative tumours (reviewed by Hawkins, 1985). A higher proportion of oestrogen receptor-positive tumours respond to anti-oestrogen therapy: paradoxically, a small number of oestrogen receptor-negative tumours also respond (Desombre *et al.*, 1978).

A variety of techniques have been used to determine oestrogen receptor protein levels in breast tumours, including tritiated hexoestrol binding *in vivo* (Folca, 1961), sedimentation assays (Desombre *et al.*, 1978), dextran-coated charcoal assays (Korenman & Dukes, 1970), radio-immunoassay (Thorpe, 1987) and immunocytochemistry (Coombes *et al.*, 1987). The recent isolation and cloning of the oestrogen receptor cDNA by Walter *et al.* (1985) has now made it possible to study oestrogen receptor mRNA expression. Parl *et al.* (1987) demonstrated oestrogen receptor mRNA in extracts of human uterus and Barrett-Lee *et al.* (1987) used dot–blots to quantify oestrogen receptor mRNA in a series of human breast tumour extracts. We describe here the detection of mRNA for the oestrogen receptor in a series of 47 breast cancer samples by hybridisation to northern transfers with a sensitive single-stranded RNA probe.

### Materials and methods

Breast tumours comprising 39 primary ductal carcinomas, 3 lymph node metastases of ductal carcinomas (one from a primary ductal carcinoma also extracted), 1 recurrent ductal carcinoma (from a primary carcinoma already extracted), 1 mucinous ductal carcinoma, 2 lobular carcinomas and 1 atypical medullary carcinoma were obtained fresh. Suitable tumour tissue for RNA extraction was selected and immediately stored in liquid nitrogen: sample weight varied from 0.3 g to 1 g. Adjacent tumour tissue was examined histologically. RNA was also prepared from combined endometrium and myometrium from the uterus of a premenopausal woman. Patient age ranged from 24 to 95 years: 14 were less than 50 years old and considered to be premenopausal.

### *RNA extraction*

RNA was extracted from the 47 tumour biopsies and the uterus using a modification of the method of Auffrey & Rougeon (1980). Tumours frozen in liquid nitrogen were reduced to a powder using a Braun mikro dismembrator. Tumour powder was transferred to a Corex centrifuge tube, suspended in 5 ml of 3 M LiCl, 6 M urea, 0.5% SDS and 50 mM sodium acetate (pH 5.6) and the DNA was sheared with an Ultraturrax homogeniser. RNA was precipitated at 4°C for 18 h and recovered by centrifugation at 10,000 *g* for 20 min. The supernatant was discarded and the resultant pellet was washed in 3 ml of 3 M LiCl, 6 M urea and 50 mM sodium acetate and recentrifuged at 10,000 *g* for 10 min. The pellet was then resuspended in 1.125 ml 10 mM Tris buffer pH 8.0, 0.2% SDS, at room temperature, Tris buffer pH 8.0 and EDTA were added to 0.1 M and 5 mM, respectively in 1.25 ml total volume and then extracted with phenol:chloroform (50:50) and chloroform:isoamyl alcohol (24:1). The organic phases were back extracted with 1.25 ml 100 mM Tris pH 9. Following the addition of 6.25 ml 100% ethanol and 50 μl 5 M NaCl, RNA was precipitated at −20°C. After centrifugation, the pellet was washed in 70% cold ethanol, recentrifuged, dried and finally redissolved in 10 mM Tris, 0.2% SDS pH 8. Total RNA yields were estimated from the optical density at 260 nm. MCF-7 cell RNA was prepared as described previously (May and Westley, 1986).

### *Electrophoresis and transfer of RNA*

RNA samples (10 μg) were denatured for 10 min at 65°C in 50% dimethylsulphoxide, 2.2 M formaldehyde, 10 mM sodium phosphate (pH 7.5), 0.5 mM EDTA and then electrophoresed through a 1.2% agarose gel containing 2.2 M formaldehyde in 10 mM sodium phosphate (pH 7.5) for 500 volt-hours. After 30 min staining in ethidium bromide the RNA was visualised by UV transillumination and the integrity of the RNA assessed. Following two 30 min equilibrations in 20 × SSC (3.0 M NaCl; 0.3 M Na Citrate) the RNA was transferred to nitrocellulose or hybond-N filters (Amersham, UK) by the method of Southern (1975). Nitro-

Correspondence: F.E.B. May.
Received 28 March 1988; and in revised form, 24 June 1988.

cellulose filters were baked under vacuum for 4h at 80°C and hybond-N filters were dried at 80°C for 30 min and then the RNA covalently fixed to the membrane by irradiation with UV light (310 nm) for 5 min.

*Probe synthesis and hybridisation*

The oestrogen receptor cDNA (pOR3) cloned in pBR 322 (Walter *et al.*, 1985) was labelled with $^{32}$P-dCTP by nick-translation to a specific activity of $5 \times 10^8$ cpm $\mu g^{-1}$. Filters were hybridised with $4 \times 10^6$ cpm $ml^{-1}$ in a solution containing 50% formamide for 3 days as described previously (Westley & May, 1984). The oestrogen receptor cDNA was subcloned into the commercially available Stratagene Bluescript vector (obtained from Northumbria Biologicals, South Nelson Industrial Estate, Cramlington, Northumberland). The multiple cloning site contained within this vector is flanked by promoters for T3 and T7 RNA polymerases. Radiolabelled probes were synthesised by transcription and hybridised at $10^7$ cpm $ml^{-1}$ to the hybond-N filters at 65°C for 72h as described previously (May & Westley, 1987). After extensive washing in $0.1 \times$ SSC at 80°C the filters were exposed to preflashed X-ray film at −70°C. The extent of hybridisation of the radiolabelled probe was determined by scanning densitometry and integration of the area under the peak. The amount of hybridised oestrogen receptor RNA was expressed in arbitrary units relative to the amount hybridising to 10 $\mu$g of MCF-7 cell total RNA.

*Oestrogen receptor assay*

For some of the tumours, the cytoplasmic oestrogen receptor content was measured in a slice of tumour adjacent to the piece from which RNA was extracted. The dextran-coated charcoal method was used with seven concentrations of tritium-labelled oestradiol 17-$\beta$ (Leake *et al.*, 1981); results were obtained by Scatchard analysis of the binding data. Tumours with oestrogen receptor levels greater than 5 fmol $mg^{-1}$ protein were considered positive. The reproducibility and quality of this assay is ascertained by participation in the British Steroid Receptor Quality Control Scheme.

### Results

*Preparation of a sensitive RNA probe for the oestrogen receptor*

The availability of cloned cDNA probes for the oestrogen receptor should allow measurement of the oestrogen receptor mRNA levels in breast tumour samples. The ability to measure oestrogen receptor mRNA in total rather than poly(A)+ RNA would facilitate routine assay by avoiding mRNA purification. To determine the sensitivity of hybridisation with a cDNA probe, the oestrogen receptor cDNA was nick-translated and hybridised to Northern transfers of 10 $\mu$g total RNA prepared from several human breast cancer cell lines known to contain the oestrogen receptor protein (Reddell *et al.*, 1985). A very weak hybridisation signal was detected to an RNA of 6.2 kb in RNA prepared from the EFM-19 cell line (Simon *et al.*, 1984); no hybridisation was detected to RNA from the MCF-7, T47D, or ZR-75 cell lines (data not shown). Therefore, to measure different levels of oestrogen receptor mRNA in total RNA preparations, it was necessary to increase the sensitivity above that obtained with the nick-translated probe.

An Eco RI fragment of the oestrogen receptor cDNA was isolated and subcloned into the Bluescript vector. The multiple cloning site contained within this vector is flanked by promoters for T3 and T7 RNA polymerases that permit transcription of high specific activity, single-stranded RNA probes from the cDNA insert (Figure 1; Melton *et al.*, 1984). As the orientation of the oestrogen receptor cDNA was unknown, we were unable to predict from which end the RNA probe should be synthesised. The construct was therefore linearised with Bam H1 and RNA transcribed from the T3 promoter (as shown in Figure 1), or with Sal 1 and RNA transcribed from the T7 promoter.

The two radiolabelled probes were hybridised to total RNA prepared from MCF-7 cells. Ten $\mu$g total RNA was electrophoresed through a denaturing agarose gel, transferred to nitrocellulose and hybridised with the probes. The autoradiographs obtained are shown in Figure 2, alongside the result obtained with the nick-translated cDNA probe for the oestrogen receptor. Strand-specific hybridisation to an RNA of 6.2 kb was obtained with the RNA initiated from the T3 promoter. This agrees with the results reported by Walter *et al.* (1985) who detected a 6.2 kb RNA in 30 $\mu$g poly A+ RNA prepared from MCF-7 and T47D cells using a nick-translated oestrogen receptor cDNA probe.

*Oestrogen receptor RNA in breast tumour specimens*

RNA was prepared from 47 breast tumour specimens and a uterus from a premenopausal woman. The weight of the tumour samples ranged from 0.3 g to 1 g and the total RNA yield varied from 54 $\mu$g to 940 $\mu$g. Agarose gel electrophoresis of these extracts revealed good quality RNA with most preparations showing only slight degradation as judged by the ratio of 28S to 18S RNA. In some instances, histologically observed tumour necrosis may explain the RNA degradation.

Ten $\mu$g of total RNA were denatured, electrophoresed on formaldehyde-containing agarose gels, transferred to a nylon membrane and hybridised with the single-stranded oestrogen receptor RNA probe. To quantify the hybridisation obtained, the autoradiographic signals were measured by scanning densitometry and integration of the area under the resultant peak. Oestrogen receptor mRNA levels were expressed as Arbitrary Units (AU), one arbitrary unit being equal to 5% of the amount of oestrogen receptor RNA present in 10 $\mu$g MCF-7 RNA. These MCF-7 cells contain ~100 fmol oestrogen receptor $mg^{-1}$ cytosolic protein.

Examples of selected results from different tissues are shown in Figure 3. Hybridisations to different concentrations of the RNA from MCF-7 cells are shown to the left of the figure and to 10 $\mu$g RNA from the uterus of a premenopausal woman on the right of the figure for comparison.

**Figure 1** Structure of Bluescript oestrogen receptor cDNA recombinant. An Eco R1 fragment of the oestrogen receptor cDNA was cloned into Bluescript (KSM13−) at the Eco R1 site. An RNA probe for the oestrogen receptor mRNA was synthesised using T3 polymerase and Bam H1 linearised DNA. The direction of RNA synthesis, the positions of 5 restriction sites in the multiple cloning site, the positions of the T3 and T7 promoters, the $\beta$-galactosidase and ampicillin resistance genes are marked.

**Figure 2** Hybridisation of oestrogen receptor probes to Northern transfers of MCF-7 RNA. $^{32}P$-labelled probes for the oestrogen receptor mRNA were synthesised by nick translation (N-T) or by transcription using T3 or T7 polymerase of the Bam H1 or Sal 1 linearised recombinant shown in **Figure 1**. The positions of the 6.2 kb oestrogen receptor mRNA and 28 and 18S ribosomal RNAs are marked.

**Figure 3** Oestrogen receptor probe hybridisation with Northern transfers of MCF-7 cells, primary ductal carcinomas of breast, breast carcinoma lymph node metastases and a uterus from a premenopausal woman. Ten μg RNA from each of the tissue samples and between 10 μg and 0.01 μg (as indicated) of the MCF-7 cell RNA were separated by gel electrophoresis, transferred to hybond-N membranes and hybridised with the single stranded oestrogen receptor RNA probe. Twenty hour and 14 day exposures are shown. The probe identified a 6.2 kb RNA species in varying quantities in all but tumour 29. The amount of oestrogen receptor RNA ranges from 0.18 AU (tumour 33) to 34.3 AU (uterus).

After an exposure time of only 20 h, a 6.2 kb RNA was clearly visible in RNA prepared from MCF-7 cells, the uterus, breast tumour 28 and the metastatic tumour in lymph node 31. After a longer exposure of 14 days, the oestrogen receptor mRNA of 6.2 kb was detected in all the samples apart from tumour 29. It is interesting that a lymph node metastasis from this tumour did express oestrogen receptor mRNA. Oestrogen receptor mRNA in these examples ranged from 0 AU (tumour 29) to 34.3 AU (uterus). Tumour 33 contained the lowest level of oestrogen receptor mRNA detected (0.18 AU): although the 6.2 kb band is barely visible on the photograph, it produced an easily quantifiable peak by scanning densitometry of the original autoradiograph.

The levels of oestrogen receptor mRNA and oestrogen receptor protein in the uterus, MCF-7 cells, the primary breast tumour samples and metastatic deposits are summarised in Table I. The quantity of oestrogen receptor mRNA detected in the 47 tumours varied from 0 to 1,993 AU. Only six tumours (13%) expressed no detectable oestrogen receptor mRNA while the other 41 (87%) expressed varying levels. Of these, 9 expressed only small amounts of oestrogen receptor mRNA (less than or equal to 1 AU, e.g. tumour 35), 26 contained intermediate amounts (between 1 and 100 AU, e.g., tumour 28) and 6 contained large quantities (in excess of 100 AU). The largest quantity (1,993 AU) was present in a lobular carcinoma: this was six times higher than the next greatest level. In one instance a primary tumour and a recurrent tumour excised from the same patient 9 months later were compared: both expressed similar low/intermediate levels of oestrogen receptor mRNA (4 AU *vs.* 1.1 AU).

### *Oestrogen receptor mRNA, tumour histology and Bloom's grade*

Thirty-nine of the tumours examined were primary ductal carcinomas of the breast. These tumours were graded with respect to differentiation using the method of Bloom & Richardson (1959): 2 tumours were grade I, 11 were grade II and 26 were grade III (this is an unusual sample, grade II normally being the predominant group). A comparison of oestrogen receptor and oestrogen receptor mRNA levels in tumours of different histological grade is shown in Table II. All 6 oestrogen receptor mRNA negative tumours were grade III and 6 of the 7 ductal carcinomas with oestrogen receptor mRNA levels of less than 1 AU were also grade III. The median of the oestrogen receptor mRNA levels was significantly lower for the grade III tumours than for the grade I and II tumours ($P < 0.05$).

Of the remaining non-ductal carcinomas, 2 were lobular carcinomas: both expressed oestrogen receptor mRNA, one expressing the highest level recorded (1,993 AU) and the

**Table I** Levels of the oestrogen receptor and its mRNA in different breast tumour samples, MCF-7 cells and a uterus.

| | *Oestrogen receptor mRNA (AU)* | *Oestrogen receptor protein (fmol mg$^{-1}$ protein)* |
|---|---|---|
| MCF-7 cells | 20 (1) | 100 (1) |
| Uterus | 34.3 (1) | ND |
| Primary tumours (No tamoxifen treatment) | 122.1 ± 61.4 (33)† | 84.3 ± 47.3 (26)† |
| Primary tumours (Tamoxifen prior to excision) | 7.8 ± 4.18 (10)† | 3.9 ± 2.4 (9)† |
| Lymph node metastases (No tamoxifen treatment) | 22.1 (2) | 47.5 (2) |
| Lymph node metastases (Tamoxifen prior to excision) | 7.2 (1) | ND |

ND = not determined; ( ) = no. samples; †mean ± s.e.m.

other expressing moderate quantities (76.7 AU). The mucinous carcinoma also expressed moderate quantities of oestrogen receptor mRNA (19.3 AU) and the atypical medullary carcinoma expressed a small amount (0.6 AU). Oestrogen receptor mRNA was present in the three lymph node metastases examined (see above).

*Menopausal status and oestrogen receptor mRNA*

The oestrogen receptor and oestrogen receptor mRNA levels in tumours from pre- and post-menopausal women are shown in Table III. There was no significant difference in median levels of oestrogen receptor mRNA between tumours from the 14 different premenopausal women (age less than 50) and the 31 postmenopausal women (Chi square = 0.437, $P > 0.5$). The percentages of tumours that express oestrogen receptor mRNA in the two groups were the same. The medians of the oestrogen receptor protein levels in tumours from premenopausal and postmenopausal women were also not significantly different (Chi square = 1.246, $P > 0.25$).

*Correlation of oestrogen receptor mRNA with oestrogen receptor protein*

For 39 samples, oestrogen receptor protein levels determined using a dextran-coated charcoal ligand binding assay were available for comparison with the mRNA levels. Using this assay any values below 5 fmol oestrogen receptor protein/mg cytosol protein were considered negative. A graphical representation of this comparison is shown in Figure 4. The oestrogen receptor protein was not detected in 21 tumours (53%) whereas the oestrogen receptor mRNA was absent in only 6 (15%). In 14 of the remaining 15 receptor protein negative tumours oestrogen receptor mRNA was present at relatively low levels (all less than 13.3 AU). The remaining member of this group of receptor protein negative tumours contained the highest level of receptor RNA detected (1,993 AU).

One tumour contained very high and two tumours relatively high levels of oestrogen receptor protein but only low levels of the mRNA. These examples may reflect tumour heterogeneity with respect to oestrogen receptor expression.

Overall, the oestrogen receptor protein levels correlated significantly with oestrogen receptor mRNA levels and Spearman's Correlation Coefficient was calculated to be 0.74, $P < 0.0001$.

*Tamoxifen and oestrogen receptor mRNA*

A number of patients had been treated with tamoxifen prior to tumour excision (Figure 4). Tamoxifen, while unlikely to affect the results of oestrogen receptor mRNA estimation, has been cited as a cause of falsely low or negative oestrogen receptor protein estimations (Hull *et al.*, 1980). In the current series, the effects of tamoxifen on receptor protein estimation was not clear cut and in only one instance was there an unexpectedly low oestrogen receptor protein level in the face of high mRNA levels. Of the remaining cases, 3 expressed low levels of oestrogen receptor mRNA but no protein (possibly reflecting the increased sensitivity of the mRNA assay), 1 expressed low levels of mRNA but high levels of protein, 2 expressed high levels of mRNA and protein, and 2 contained no RNA or protein. Overall, there were not significantly fewer oestrogen receptor positive tumours in the group of patients treated with tamoxifen (Chi squared test; $P > 0.1$).

### Discussion

In this paper we describe the isolation of high quality RNA and subsequent detection of oestrogen receptor mRNA in a series of breast tumours. We detected hybridisation to a single oestrogen receptor RNA band of approximately 6.2 kb in extracts of RNA prepared from MCF-7 breast cancer cells, all the positive breast tumours and a uterus from a premenopausal woman. A 6.2 kb RNA was detected previously in MCF-7 cell RNA (Walter *et al.*, 1985) whereas others detected a 4.2 kb oestrogen receptor RNA in the uterus from a premenopausal woman (Parl *et al.*, 1987). We did not detect the 3.7 kb oestrogen receptor RNA found by Barrett-Lee *et al.* (1987) in both MCF-7 cell and breast tumour RNA.

Estimation of the level of the oestrogen receptor mRNA in Northern transfers of tumour extracts is a highly sensitive method of measuring oestrogen receptor mRNA levels. The presence of a single, well-defined hybridisation band of 6.2 kb is unequivocal evidence of the presence of oestrogen receptor mRNA and problems of non-specific background hybridisation are largely eliminated, allowing confident determination, even at low levels of oestrogen receptor expression. The notoriously unstable nature of mRNA does present problems, but RNA degradation was minimised by prompt handling and cryo-preservation of tumour tissue, coupled with scrupulous preparation of glassware, chemicals and instruments.

Measurement of oestrogen receptor mRNA levels was more sensitive than the ligand binding oestrogen receptor protein assays in this study. Great variation in the proportion of tumours expressing the oestrogen receptor protein has been reported (Thorpe, 1987). Of the 45 breast tumours from different patients included in this study, 39 (87%) contained detectable quantities of oestrogen receptor mRNA. This figure is even more remarkable when the large numbers

**Table II** Comparison of oestrogen receptor and oestrogen receptor mRNA levels in tumours graded according to Bloom and Richardson (1959).

| | *Oestrogen receptor mRNA (AU)* | | | *Oestrogen receptor protein (fmol mg$^{-1}$ protein)* | | |
|---|---|---|---|---|---|---|
| | *Mean* | *Median* | *No.* | *Mean* | *Median* | *No.* |
| Grade I and II | 67.9 ± 28.5 | 32.7[a] | 13 | 79.2 ± 63.2 | 7.2 | 8 |
| Grade III | 43.6 ± 19.9 | 1.4[a] | 26 | 67.6 ± 49.9 | 0 | 21 |

[a]$P < 0.05$.

**Table III** Comparison of oestrogen receptor and oestrogen receptor mRNA levels in tumours from premenopausal and postmenopausal women.

| | *Oestrogen receptor mRNA (AU)* | | | *Oestrogen receptor protein (fmol mg$^{-1}$ protein)* | | |
|---|---|---|---|---|---|---|
| | *Mean* | *Median* | *No.* | *Mean* | *Median* | *No.* |
| Premenopausal | 63.6 ± 30.7 | 20.7 | 14 | 87.4 ± 45.9 | 39 | 11 |
| Postmenopausal | 105.4 ± 64.8 | 64.8 | 31 | 52.8 ± 49.9 | 0 | 26 |

**Figure 4** Correlation between oestrogen receptor mRNA and protein levels. □ = patients pretreated with tamoxifen. mRNA and protein levels were compared using Spearman's Correlation Coefficient, $r_s = 0.74$, $P < 0.0001$, $n = 39$.

of poorly differentiated (grade III) tumours included in this study are considered. In the 37 different tumours where the levels of the oestrogen receptor and mRNA were compared, using 5 fmol oestrogen receptor protein/mg cytosolic protein as the cut off for receptor positivity, only 46% were oestrogen receptor protein positive. This reflects a genuine difference in sensitivity. The incidence of oestrogen receptor positivity, although low in comparison to some other studies, can be attributed to the following features of the population studied: the disproportionately high number of grade III tumours; selection of patients aged over 70 on grounds of failure to respond to tamoxifen (patients in this group who responded to tamoxifen were not treated surgically hence reducing the number of likely oestrogen receptor positive subjects); and selection of material principally from larger tumours (only these tumours yielded sufficient tissue for both assays).

The functional significance of the expression of low levels of oestrogen receptor remains to be established, particularly with regard to determining response to antioestrogen therapy. Such low (and previously undetectable) levels of oestrogen receptor may account for the seemingly anomalous remission following antioestrogen therapy seen in 5 to 10% of apparently oestrogen receptor-negative breast tumours (McGuire *et al.*, 1975). Low levels of oestrogen receptor expression may be due to uniform low levels of oestrogen receptor expression throughout a tumour or may reflect tumour heterogeneity, with small areas of otherwise negative tumours expressing high levels of oestrogen receptor. This issue remains to be settled although there is evidence to support the latter contention (van Netten *et al.*, 1985): high resolution *in situ* hybridisation techniques using sensitive probes may be of value. There is also heterogeneity of oestrogen receptor expression between primary tumours and their lymph node metastases. Extracts of one primary breast tumour (tumour 29, figure 3) contained no oestrogen receptor mRNA, yet a lymph node metastasis from the same tumour did. Similar disparities between the oestrogen receptor levels in primary tumours and lymph node metastases have been reported previously (Rosen *et al.*, 1977), although the converse situation arises more frequently (Castagnetta *et al.*, 1987). Such heterogeneity may also explain the objective response to antioestrogen therapy seen in a small proportion of apparently oestrogen receptor-negative tumours.

One of the two lobular carcinomas examined expressed extremely high levels of oestrogen receptor mRNA (1,993 AU) in the absence of detectable oestrogen receptor protein (Figure 4). While this may be explained by extreme tumour heterogeneity, a further intriguing possibility is that in this tumour the oestrogen receptor gene is transcribed but the RNA is not translated. The exceptionally high level of oestrogen receptor mRNA may be due to loss of some normal feedback mechanism dependent on synthesis of receptor protein.

Oestrogen receptor mRNA expression was also considered with regard to tumour histology, differentiation and menopausal status. Oestrogen receptor mRNA was detected in all of the histological sub-types examined, in agreement with the receptor protein findings of Rosen *et al.* (1978). When tumour differentiation was considered all the oestrogen receptor mRNA negative ductal carcinomas and all but one of those expressing only very low levels of oestrogen receptor mRNA (<1 AU) belonged to grade III of Bloom & Richardson's classification (1959): the median of the oestrogen receptor mRNA levels was significantly lower in this group (Table II). This trend is in agreement with other studies of oestrogen receptor protein expression (McCarty *et al.*, 1980, Furmanski *et al.*, 1980, Hawkins *et al.*, 1987). As oestrogen receptor expression is a differentiated feature of breast epithelial cells, it is attractive to hypothesise that its expression should decline with loss of morphological features of differentiation. Median oestrogen receptor mRNA and protein levels were similar in tumours from both premenopausal and postmenopausal women: other studies of protein levels (Clark *et al.*, 1983; Hawkins *et al.*, 1987) and mRNA levels (Barrett-Lee *et al.*, 1987) found higher levels in tumours from post-menopausal patients. The reason for the differences between these studies and the present work are not clear.

Determination of the oestrogen receptor mRNA content using Northern transfers of tumour RNA offers a new approach to the investigation of oestrogen receptor expression and status in breast cancer. This technique is highly sensitive and oestrogen receptor may be detectable in a higher proportion of breast tumours than previously thought. The biological significance of low levels of oestrogen receptor expression remains to be determined.

We thank Prof P. Chambon for oestrogen receptor cDNA. J.A. Henry thanks the Wellcome Trust for a research training fellowship. F.E.B. May is a recipient of 1983 University Research Fellowship from the Royal Society. S. Cousen, R. Brown and P. Chambers provided invaluable technical assistance.

### References

AUFFRAY, C. & ROUGEON, F. (1980). Purification of mouse immunoglobulin heavy-chain messenger RNAs from total myeloma tumour RNA. *Eur. J. Biochem.*, **107**, 303.

BARRETT-LEE, P.J., TRAVERS, M.T., McCLELLAND, R.A., LUQMANI, Y. & COOMBES, R.C. (1987). Characterisation of estrogen receptor messenger RNA in human breast cancer. *Cancer Res.*, **47**, 6653.

BLOOM, H.J.G. & RICHARDSON, W.W. (1959). Histological grading and prognosis in breast cancer: a study of 1,409 cases of which 359 have been followed for 15 years. *Br. J. Cancer*, **11**, 359.

CASTAGNETTA, L., TRAINA, A., DI CARLO, A., LATTERI, A.M., CARRUBA, G. & LEAKE, R.E. (1987). Heterogeneity of soluble and nuclear oestrogen receptor status of involved nodes in relation to primary breast cancer. *Eur. J. Cancer Clin. Oncol.*, **23**, 31.

CLARK, G. & McGUIRE, W.L. (1983). Estrogen receptor content of human breast cancer tumors is related to age independently of menopausal status but progesterone receptor is related to both age and menopausal status. *Proc. Amer. Soc. Clin. Oncol.*, **2**, 392 (*abstract*).

COOMBES, R.C., POWLES, T.J., BERGER, U. & 5 others (1987). Prediction of endocrine response in breast cancer by immunocytochemical detection of oestrogen receptor in fine needle aspirates. *Lancet*, **ii**, 701.

DESOMBRE, E.R., GREENE, G.L. & JENSEN, E.V. (1978). Estrophilin and endocrine responsiveness of breast cancer. In *Hormones, receptors and breast cancer*, McGuire, W.L. (ed) p. 1. Raven Press: New York.

FOLCA, P.J., GLASCOCK, R.F. & IRVINE, W.T. (1961). Studies with tritium labelled hexoesterol in advanced breast cancer. *Lancet*, **ii**, 796.

FURMANSKI, P., SAUNDERS, D.E., BROOKS, S.C. & THE BREAST CANCER PROGNOSTIC STUDY CLINICAL AND PATHOLOGY ASSOCIATES (1980). The prognostic value of estrogen receptor determination in patients with primary breast cancer – an update. *Cancer*, **46**, 2794.

HAWKINS, R.A. (1985). Receptors in the management of breast cancer. *Br. J. Hosp. Med.*, **34**, 160.

HAWKINS, R.A., WHITE, G., BUNDRED, N.J. & 4 others (1987). Prognostic significance of oestrogen and progestogen receptor activities in breast cancer. *Br. J. Surg.*, **74**, 1009.

HULL, D.F., CLARK, G.M., OSBORNE, C.K., CHAMNESS, G.C., KNIGHT, W.A. & McGUIRE, W.L. (1980). Multiple estrogen receptor assays in human breast cancer. *Cancer Res.*, **43**, 413.

KORENMAN, S.G. & DUKES, B.A. (1970). Specific estrogen binding by the cytoplasm of human breast carcinoma. *J. Clin. Endocrinol. Metab.*, **30**, 639.

LEAKE, R.E., LAING, L., CALMAN, K.C., MACBETH, F.R., CRAWFORD, D. & SMITH, D.C. (1981). Oestrogen receptor status and endocrine therapy of breast cancers: Response rates and status stability. *Br. J. Cancer*, **43**, 59.

McCARTY, K.S., BARTON, T.K., & FETTER, B.F. (1980). Correlation of estrogen and progesterone receptors with histologic differentiation in mammary carcinoma. *Cancer*, **46**, 2851.

McGUIRE, W.L., CARBONNE, P.D. & VOLLMER, R.P. (eds) (1975). *Estrogen receptor and human breast cancer*. Raven Press: New York.

MAY, F.E.B. & WESTLEY, B.R. (1986). Cloning of estrogen-regulated messenger RNA sequences from human breast cancer cells. *Cancer Res.*, **46**, 6034.

MELTON, D.A., KREIG, P.A., REBAGLIATI, M.R., MANIATIS, T., ZINN, K. & GREEN, M.R. (1984). Efficient *in vitro* synthesis of biologically active RNA and RNA hybridisation probes from plasmids containing a bacteriophage SP6 promoter. *Nucl. Acids Res.*, **12**, 7035.

PARL, F.F., SCHONBAUM, C.P., COX, D.L. & CAVENER, D.R. (1987). Detection of estrogen receptor mRNA in human uterus. *Mol. Cell. Endocrinol.*, **52**, 235.

REDDELL, R.R., MURPHY, L.C., HALL, R.E. & SUTHERLAND, R.L. (1985). Differential sensitivity of human breast cancer cell lines to the growth inhibitory effects of tamoxifen. *Cancer Res.*, **45**, 1525.

ROSEN, P.P., MENENDEZ-BOTET, C.J., SENIE, R.T., SCHWARTZ, M.K., SCHOTTENFELD, D. & FARR, G.H. (1978). Estrogen receptor protein (ERP) and the histopathology of human mammary carcinoma. In *Hormones, Receptors and Breast Cancer*, McGuire, W.L. (ed) p. 71. Raven Press: New York.

ROSEN, P.P., MENENDEZ-BOTET, C.J., URBAN, J.A., FRACCHIA, A. & SCHWARTZ, M.K. (1977). Estrogen receptor protein (ERP) in multiple tumour specimens from individual patients with breast cancer. *Cancer*, **39**, 2194.

SIMON, W.E., ALBRECHT, M., TRAMS, G., DIETEL, M. & HOLZEL, F. (1984). *In vitro* growth promotion of human mammary carcinoma cells by steroid hormones, tamoxifen, and prolactin. *J. Natl Cancer Inst.*, **73**, 313.

SOUTHERN, E.M. (1975). Detection of specific sequences among DNA fragments separated by gel electrophoresis. *J. Mol. Biol.*, **98**, 503.

THORPE, S.M. (1987). Monoclonal antibody technique for detection of estrogen receptors in human breast cancer: greater sensitivity and more accurate classification of receptor status than the dextran-coated charcoal method. *Cancer Res.*, **47**, 6572.

VAN NETTEN, J.P., ALGARD, F.T., COY, P. & 6 others (1985). Heterogenous estrogen receptor levels detected via multiple microsamples from individual breast cancers. *Cancer*, **56**, 2019.

WALTER, P., GREEN, S., GREENE, G. & 8 others (1985). Cloning of the human estrogen receptor cDNA. *Proc. Natl Acad. Sci. USA*, **82**, 7889.

WESTLEY, B. & MAY, F.E.B. (1984). The human genome contains multiple sequences of varying homology to mouse mammary tumour virus DNA. *Gene*, **28**, 221.

WESTLEY, B.R. & MAY, F.E.B. (1987). Oestrogen regulates cathepsin D mRNA levels in oestrogen responsive human breast cancer cells. *Nucl. Acids Res.*, **15**, 3773.